# Supplementary material for: Deforestation-induced drying lowers Amazon climate threshold
Source: Nature. 2026 May 6;654(8117):114–20. doi: 10.1038/s41586-026-10456-0 (PMC13233621; doi:10.1038/s41586-026-10456-0)
Supplement: Supplementary file 1 — Supplementary analyses (Supplementary Figs. 2–6 and Supplementary Figs. 8–18) and graphical representations of the deforestation/land-use-change data basis that led to the results of our work (Supplementary Figs. 1 and 7) and Supplementary Notes. [file 41586_2026_10456_MOESM1_ESM.pdf]

nature portfolio

https://doi.org/10.1038/s41586-026-10456-0

Supplementary information

Deforestation-induced drying lowers Amazon climate threshold

In the format provided by the authors and unedited

Supplementary Information

of

Deforestation-induced drying lowers Amazon climate threshold

Nico Wunderling, Boris Sakschewski, Johan Rockström,
Bernardo M. Flores, Marina Hirota, Arie Staal

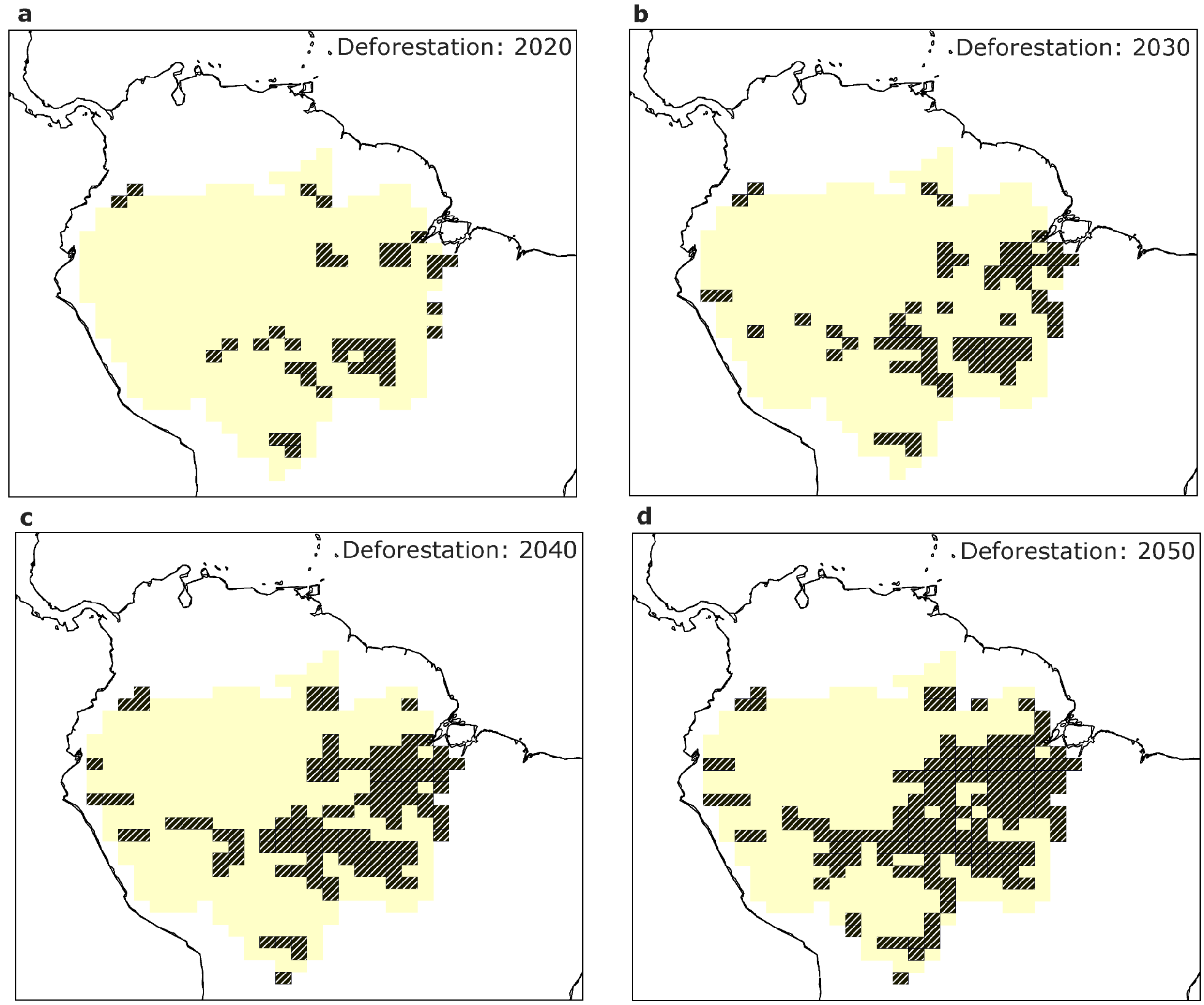

Figure S1 | Regional distribution of Business-as-Usual deforestation scenario. Business-as-Usual deforestation where the deforestation exceeds 50% of the grid cell (black hatched areas) following Soares-Filho et al. (2013)[1] in **a,** the year 2020, **b,** the year 2030, **c,** the year 2040 and **d,** the year 2050. After the year 2050, the deforestation is kept constant until the year 2099. The deforestation strongly increases from around 20% of the Amazon basin (panel **a**) until close to 40% (panel **b**). In all our simulations including deforestation, we use the exact percentage value of deforestation as input value on a grid cell basis.

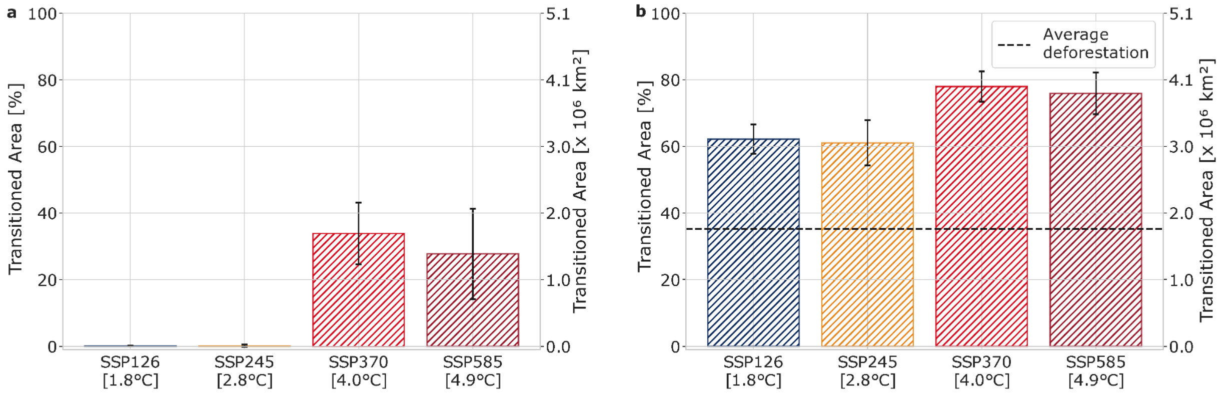

Figure S2 | Transition responses in the Amazon forest system with respect to global warming and deforestation in the 2090ies. a, Transitioned area in percent and km² of the entire Amazon basin in the 2090ies (average from 2090-2099) without deforestation. The transitioned area is very low in SSP2-4.5 (representing 2.8°C of global warming) but becomes evident for stronger climate change scenarios SSP3-7.0 (representing 4.0°C of global warming). **b,** Same as in panel a but with deforestation. Under the BaU deforestation, all scenarios show large transitioning risks. The presented mean values (and error bar values) are the spatial average over the Amazon basin consisting of 416 grid cells. Of these 416 grid cell mean values (and error bar values), each is constructed from the mean (and the standard deviation) of ten ensemble members (see methods: Ensemble construction). This notion of presented mean values and error bars is also applied to supplementary Figs. S3, S4, S5 a, c-e, and Figs. S8 and S9.

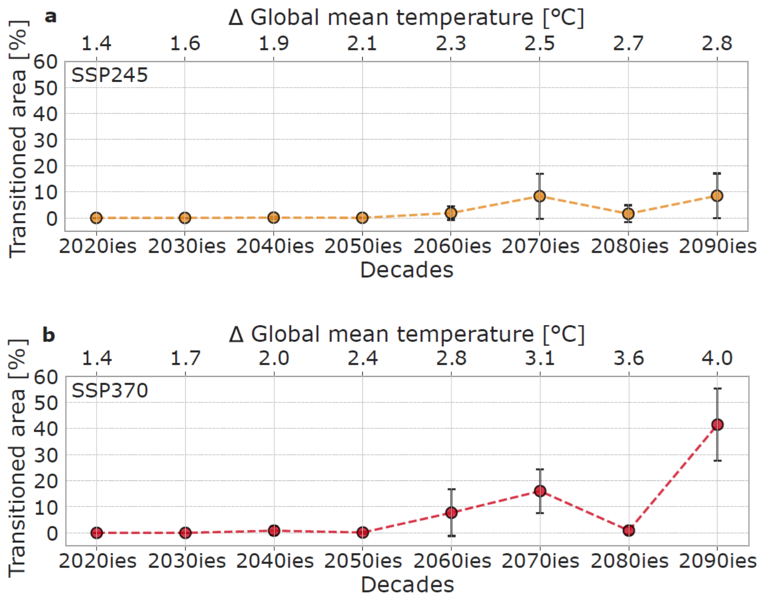

Figure S3 | Transitioned area for MAP as sole critical variable. a, Transitioned area in percent for each decade starting from the 2020ies up to the 2090ies for SSP2-4.5. **b,** Same for SSP3-7.0. The presented mean values (and error bar values) are the spatial average over the Amazon basin consisting of 416 grid cells (see also caption of supplementary Fig. S2).

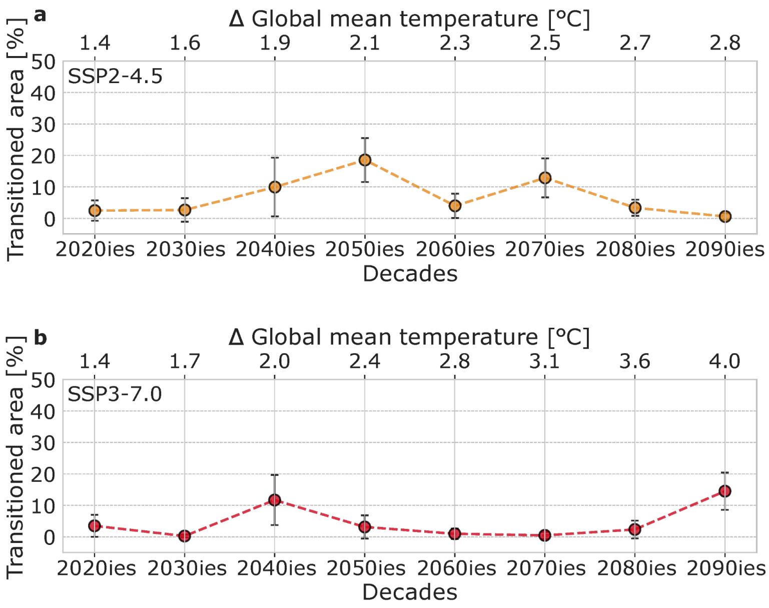

Figure S4 | Transitioned area for MCWD as sole critical variable. a, Transitioned area in percent for each decade starting from the 2020ies up to the 2090ies for SSP2-4.5. **b,** Same for SSP3-7.0. The presented mean values (and error bar values) are the spatial average over the Amazon basin consisting of 416 grid cells (see also caption of supplementary Fig. S2).

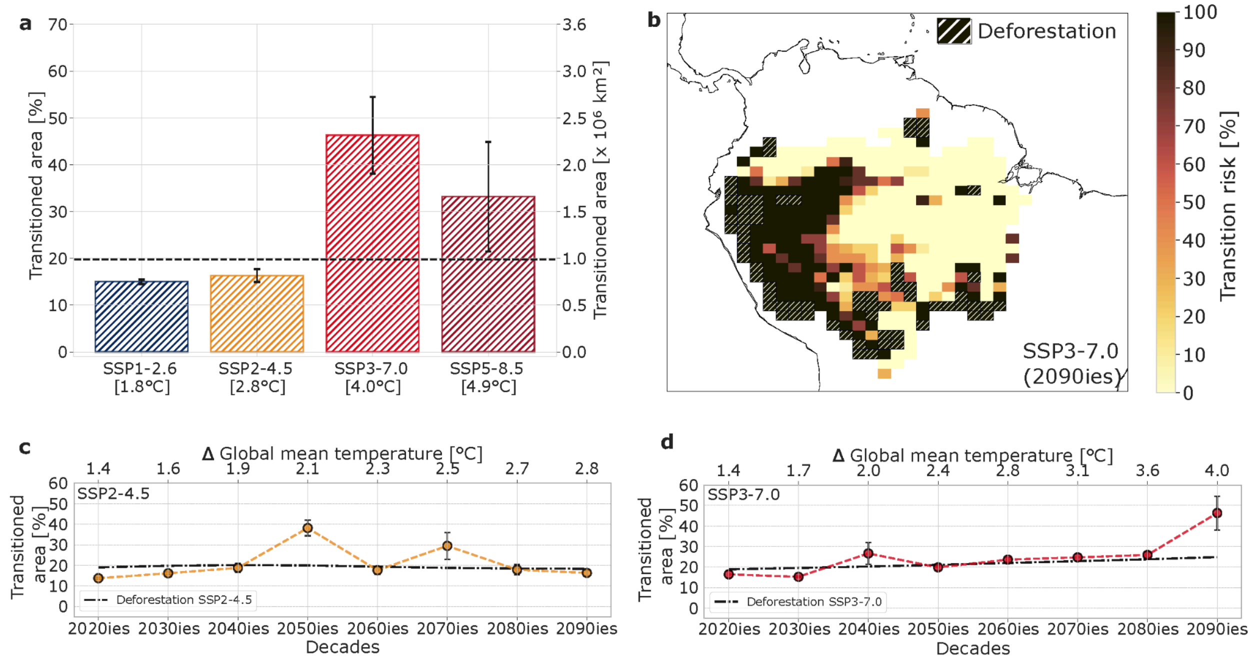

Figure S5 | Effect of SSP-based deforestation scenarios on the transitioned area in the Amazon forest. **a,** Transitioned area in percent and km² of the entire Amazon basin with a sharp increase between an SSP2-4.5 (2.8°C of global warming) and SSP3-7.0 (4.0°C of global warming) scenario in the 2090ies. **b,** Regions of the Amazon forest most at risk of crossing critical thresholds (west and southwestern part of the Amazon basin) for an SSP3-7.0 scenario at 4.0°C (2090ies decade) of global warming. The hatched region denotes deforested locations. **c,** Transitioned area in percent for each decade starting from the 2020ies up to the 2090ies for SSP2-4.5 including deforestation for the respective decade (black dash-dotted line). **d,** Same for SSP3-7.0. The presented mean values (and error bar values) are the spatial average over the Amazon basin consisting of 416 grid cells (see also caption of supplementary Fig. S2).

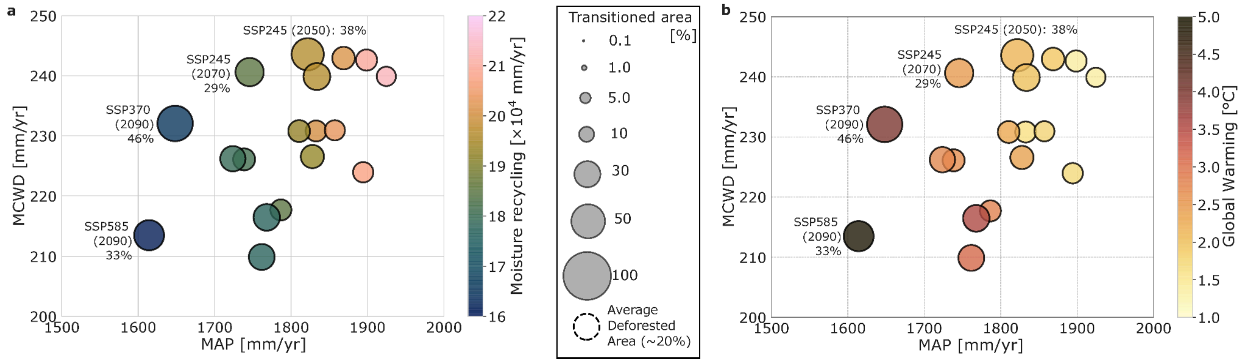

Figure S6 | Critical thresholds in annual water supply (MAP, MCWD) for SSP deforestation scenarios. a, Transitioned area (size of the circle; in percent of the entire Amazon basin) dependent on the MAP and MCWD. The color of the circle depicts the moisture recycling strength across the entire Amazon basin. **b,** Same as in panel a but for the respective global warming levels. The circles are larger than without deforestation due to the deforested parts of the Amazon forest (~20%, see size of deforested circle as black dashed circular line).

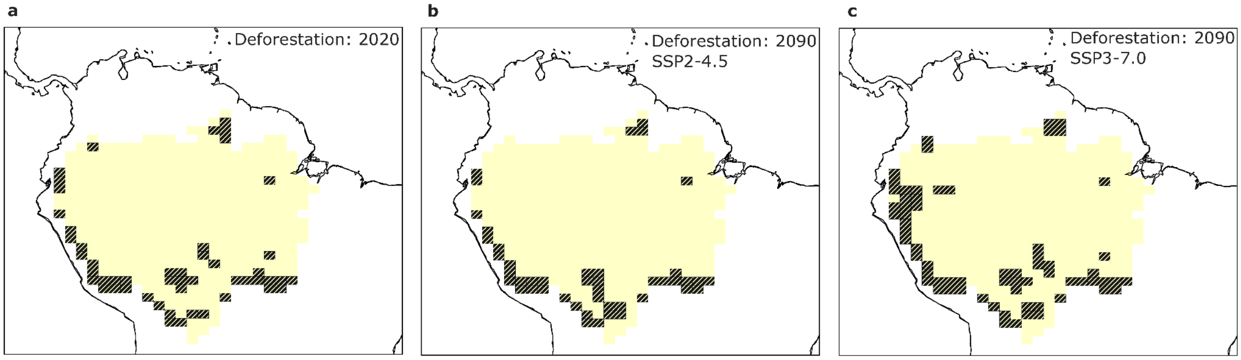

Figure S7 | Regional distribution of SSP-based deforestation scenario. SSP-based land-use changes (i.e. deforestation) where the deforestation exceeds 50% of the grid cell (black hatched areas) in **a,** the year 2020, **b,** the year 2090 for SSP2-4.5, **c,** the year 2090 for SSP3-7.0. In these scenarios, deforestation only weakly increases from around 20% to 25% for SSP3-7.0 of the Amazon basin (panel **c**). For SSP2-4.5, deforestation remains at 20% across the 21st century and is even declining towards the end of the century (panel **b**).

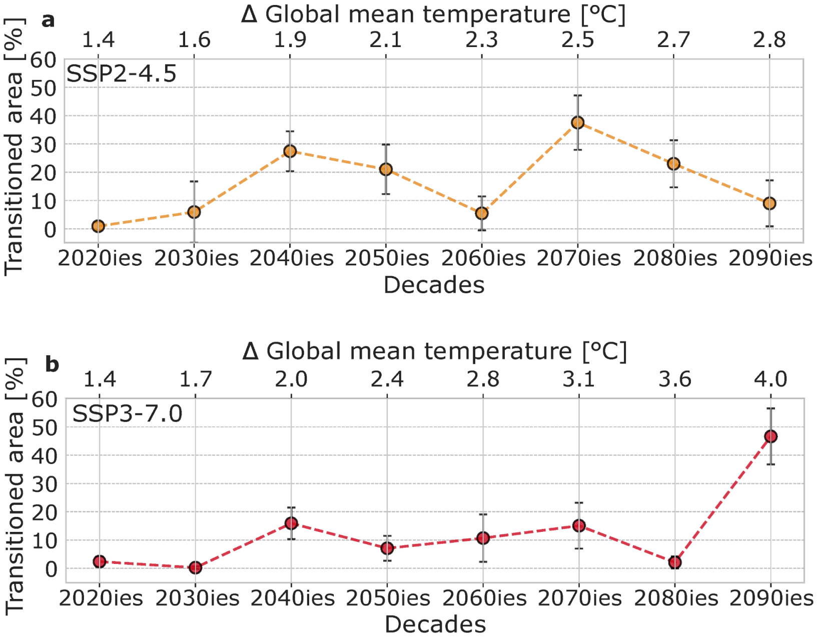

Figure S8 | Transitioned area for adaptive and fixed critical thresholds. Following Flores et al., (2024)[2], we quantify the transitioned area across the 21st century for **a,** SSP2-4.5 and **b,** SSP3-7.0. This robustness check shows that SSP3-7.0 transition risks, again, strongly increase for the decade 2090. More details on the setup of this robustness check, see methods: *robustness checks*. The presented mean values (and error bar values) are the spatial average over the Amazon basin consisting of 416 grid cells (see also caption of supplementary Fig. S2).

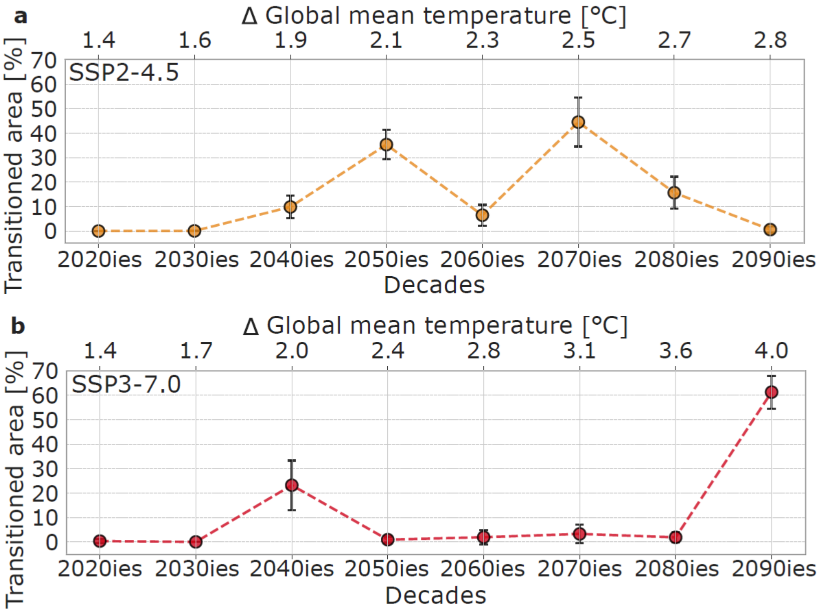

Figure S9 | Transitioned area for constant evapotranspiration of 100mm/month. Transitioned area across the 21st century for **a,** SSP2-4.5 and **b,** SSP3-7.0. This robustness check shows that SSP3-7.0 transition risks, again, strongly increase for the decade 2090. More details on the setup of this robustness check, see methods: *robustness checks*. The presented mean values (and error bar values) are the spatial average over the Amazon basin consisting of 416 grid cells (see also caption of supplementary Fig. S2).

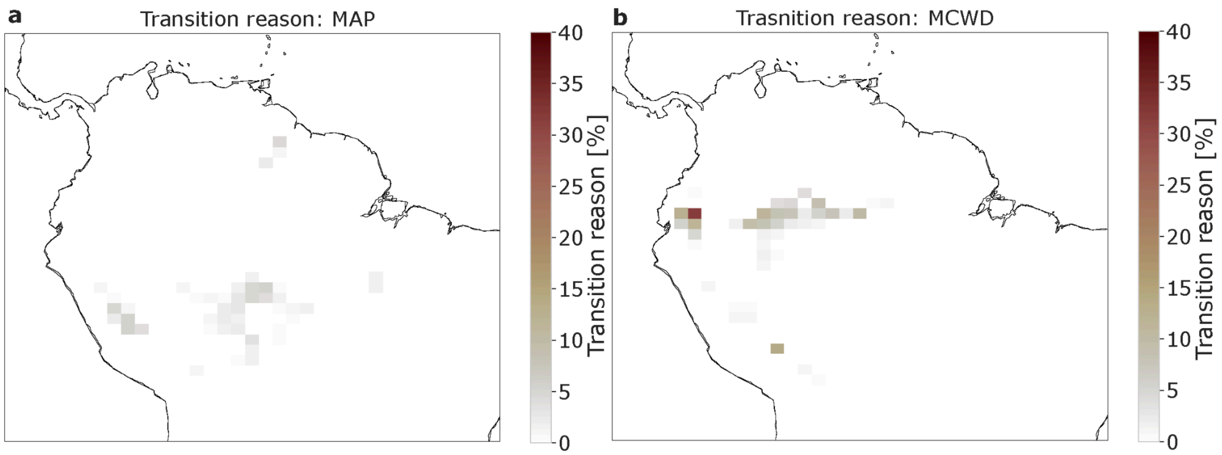

Figure S10 | Transition reason across all investigated emission scenarios. a, Transition reason: MAP. **b,** Transition reason: MCWD.

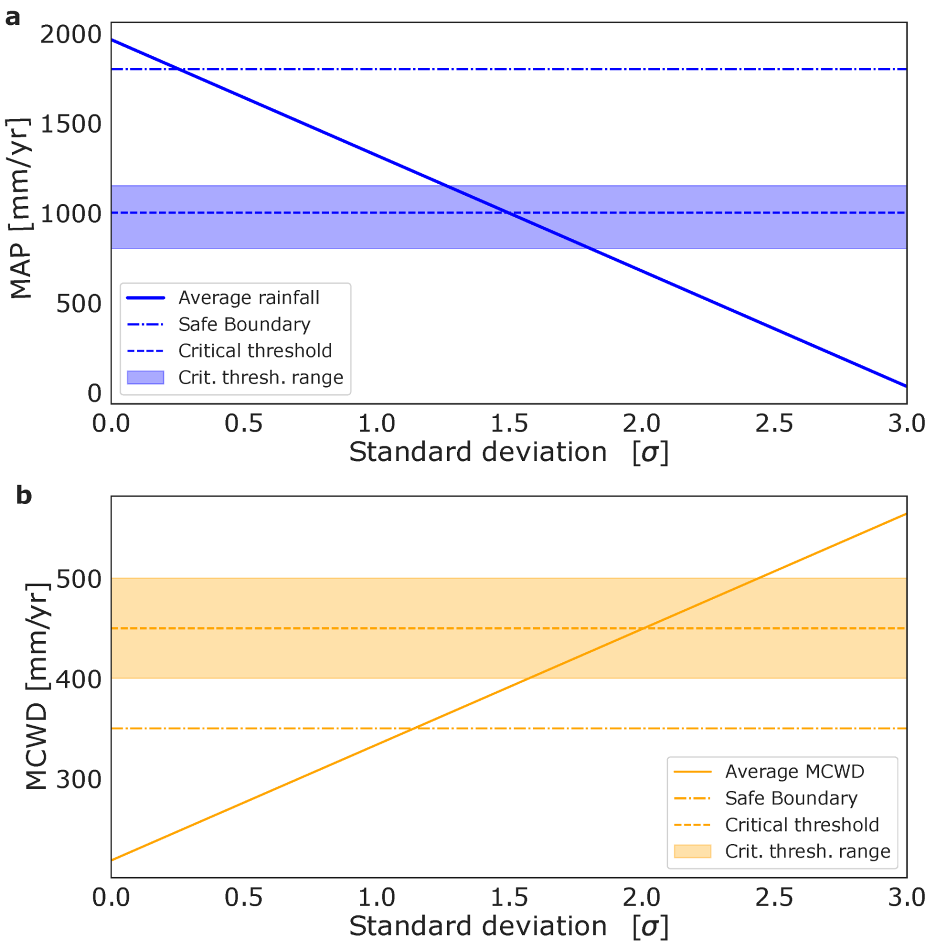

Figure S11 | Safe boundaries of MAP and MCWD for experiments with local adaptations and fixed critical thresholds. a, The solid line shows the basin-wide average for MAP in dependence of the standard deviation. At $\sigma = 0.0$, the average precipitation is around 2000 mm/yr. For $\sigma = 1.0$, the solid line denotes the MAP minus one standard deviation, etc.. The dash-dotted line shows the safe boundary (following Flores et al. (2024)[2]) above which there is no transition allowed in the experiments with local adaptations and fixed critical thresholds, and the dashed line (and shading) the critical threshold and its ranges. **b,** Same as in panel a but for MCWD.

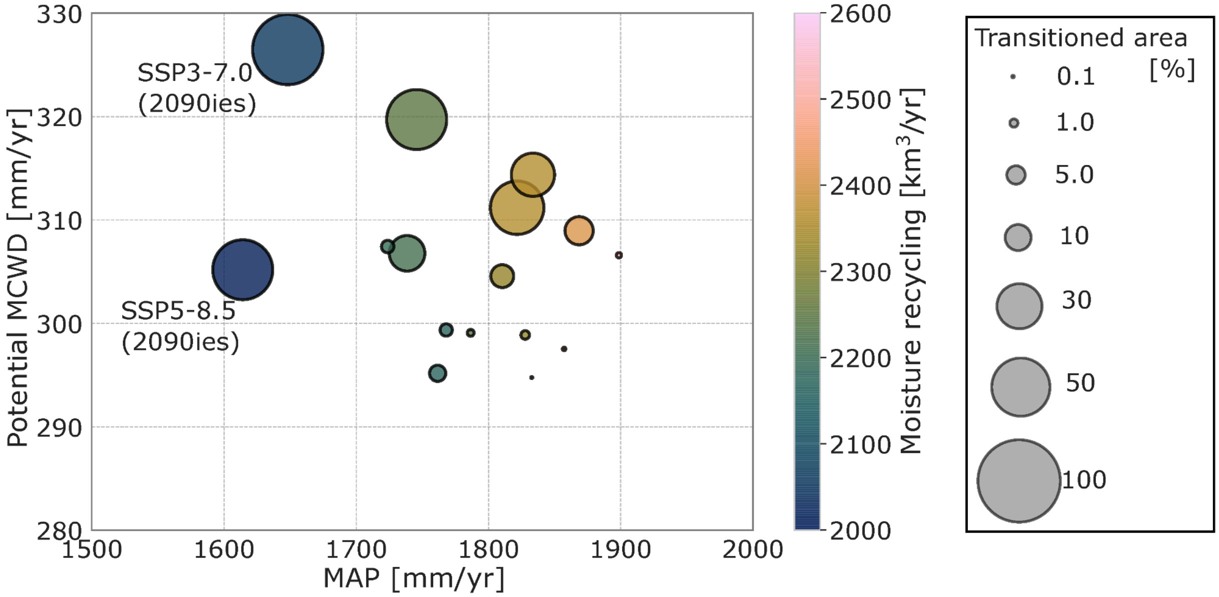

Figure S12 | Critical thresholds in annual water supply (MAP, MCWD) for constant evapotranspiration of 100 mm/month. Transitioned area (size of the circle; in percent of the entire Amazon basin) dependent on the MAP and MCWD. The color of the circle depicts the moisture recycling strength across the entire Amazon basin.

Supplementary Note: Validation of moisture recycling based on ERA5 reanalysis data

We validated our moisture tracking runs using historical ERA5 reanalysis data from 2015-2019, the period prior to that of our main results with temporal overlap between ScenarioMIP and ERA5. First, we performed monthly forward-tracking runs for the Amazon using the ERA5-based UTrack model[3] and recorded the basin precipitation recycling ratios. Second, because wind speeds may affect cascading transitions via moisture transfer, we analyzed horizontal wind speeds in ERA5 and compared them to the wind speeds in NorESM2. These wind speeds were weighted by the specific humidity profile along the vertical atmospheric column, to more accurately estimate the horizontal moisture transport and differences between ERA5 and NorESM2 therein. From ERA5 we determined daily average wind speeds, the wind speeds at 00UTC, and the daily variation in wind speeds. From NorESM2 we took daily wind speeds from SSP2-4.5 (the scenario closest to the current emission pathway[4]), years 2015-2019, for comparison with ERA5. Below we describe the methods and results of these validations.

Precipitation recycling ratios

We did monthly forward-tracking runs of UTrack[3] for the Amazon for 2015-2019 and compared the basin precipitation recycling ratios (the fraction of precipitation that last evaporated from the Amazon itself) with those from the NorESM2-based runs. For this, we performed additional runs with the NorESM2-based model for this period in SSP2-4.5. Due to the different forcing data, some differences in model setup were chosen compared to the runs for the main results of this study. In its original version, UTrack is forced by hourly ERA5 reanalysis data and, therefore, has time steps of 0.25 hours rather than 4 hours in this NorESM2-based study. For consistency, we set the time step of the NorESM2-forced validation runs to 0.25 hours as well. Also, in contrast to the runs for the main results, which involved separate forward-tracking runs for each grid cell, the moisture parcels were released across the Amazon at each time step. Following recent literature by Staal et al.[5, 6], these 100 moisture parcels were released for each mm of evapotranspiration averaged across the source area (here, the Amazon basin), in which each parcel is assigned a random initial position with a probability that scales with the spatial distribution of evapotranspiration at the respective time step. Thus, a realistic spatial distribution of moisture parcels was ensured. We recorded the precipitation that originated from within the basin and calculated the respective recycling ratios for 2015-2019. These data were regridded from 0.25° to 1° and the nearest neighbors of each of the NorESM2 grid cell coordinates was taken. The recycling ratios are on average 1.6 percentage points (5.5%) higher in NorESM2 than in ERA5. They show good correspondence and are plotted against each other in Fig. S13.

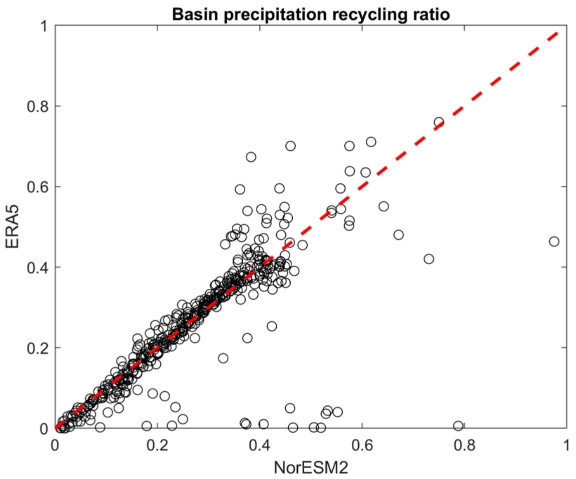

Figure S13 | Precipitation recycling ratios (-) in the Amazon during 2015-2019 based on ERA5 against NorESM2. The data points represent the 416 cells on the NorESM2 grid. The dashed line represents y=x. The average basin precipitation recycling ratio from NorESM2 is 0.27 and the average from ERA5 is 0.26. A linear regression gives R^2 = 0.59.

Weighted wind speeds

Since wind speed variabilities[7] and biases may have a significant impact on our results, we compared the daily wind speeds for 2015-2019 in ERA5 and NorESM2 (SSP2-4.5). We took the average wind speeds across the (25 and 8) pressure layers, in which we weighted these layers by their specific humidity on the respective day. As with the precipitation recycling ratios, we regridded the ERA5 results to 1° and took from these the nearest neighbors of each of the NorESM2 grid cells. The weighted wind speeds in ERA5 are 7.9% higher than in NorESM2. In Fig. S14, these ERA5 and NorESM2 wind speeds are plotted against each other.

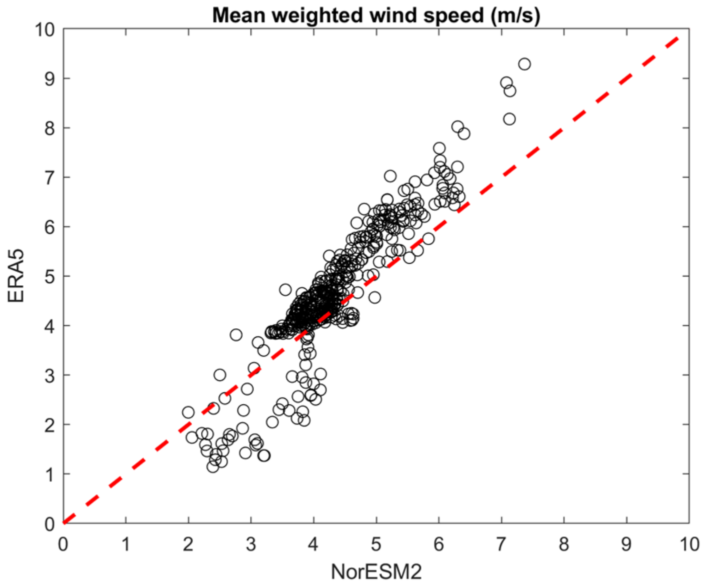

Figure S14 | Daily mean humidity-weighted wind speeds in the Amazon during 2015-2019 in ERA5 and NorESM2. The data points represent the 416 cells on the NorESM2 grid. The dashed line represents y=x. The mean wind speed in NorESM2 is 4.3 m/s and the mean in ERA5 is 4.7 m/s. A linear regression gives $R^2 = 0.84$.

An important difference between the two data sources is that ERA5 has hourly data whereas NorESM2 is based on daily data at 00UTC. Potentially, this may lead to bias in the cascading transitions, if winds at 00UTC tend to either under- or overestimate daily average winds. To explore this, we related the daily average humidity-weighted wind speeds in ERA5 against those at 00UTC. The two have high correspondence with $R^2 = 0.99$, and the wind speeds at 00UTC across the Amazon underestimate the daily averages by less than 3% (Fig. S15).

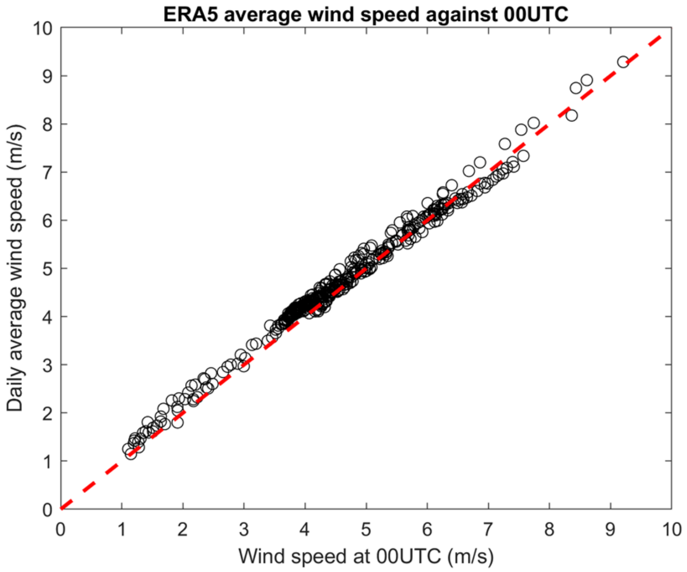

Figure S15 | ERA5-based daily average humidity-weighted wind speed in the Amazon against the wind speed at 00UTC. The data points represent the 416 cells on the NorESM2 grid. The dashed line represents y=x. The mean wind speed is 4.7 m/s and that at 00UTC is 4.5 m/s. A linear regression gives $R^2 = 0.99$.

Because of the potential role of sub-daily moisture fluxes in the Amazon, we also explored the relation between daily wind speed in ERA5 and the within-day variations in wind speed. As expected, we found that daily average wind speed positively correlates to the daily variation in wind speed, as defined by the maximum hourly wind speed minus the minimum hourly wind speed in a day, with $R^2 = 0.60$. However, there was no significant correlation ($p = 0.35$) between daily wind speed and the coefficient of variation of hourly wind speeds in a day. Therefore, we conclude that there is likely no systematic bias that is caused by the use of daily wind speed data as compared to sub-daily wind speed data.

Transition risks and wind speed changes

Moving beyond the comparison of NorESM2 with ERA5, we analyzed the wind speed changes across the 21st century in order to explore how they relate to the estimated transition risks. If a strong relation exists, it may suggest an important role of wind speed in our main results, pointing to a potentially important source of bias. For this we take the change in humidity-weighted daily wind speeds in the Amazon for 2091-2100 for SSP2-4.5, SSP3-7.0 and SSP5-8.5 compared to 2021-2030 for SSP2-4.5. We relate these changes to the transition risks in the respective scenarios and decade. We find that across SSPs, the relation between wind speed change and transition risk is significantly positive ($R^2 = 0.33$) (see Fig. S16). However, the spatial correspondence between tipping risk and wind speed changes is weak (Fig. S17), indicating that wind speed changes are not the primary cause of tipping risk. Together with the absence of clear bias in wind speeds as indicated by Figs. S13-S18, we rule out that our findings about transition (tipping points) are caused by bias in wind speeds in NorESM2.

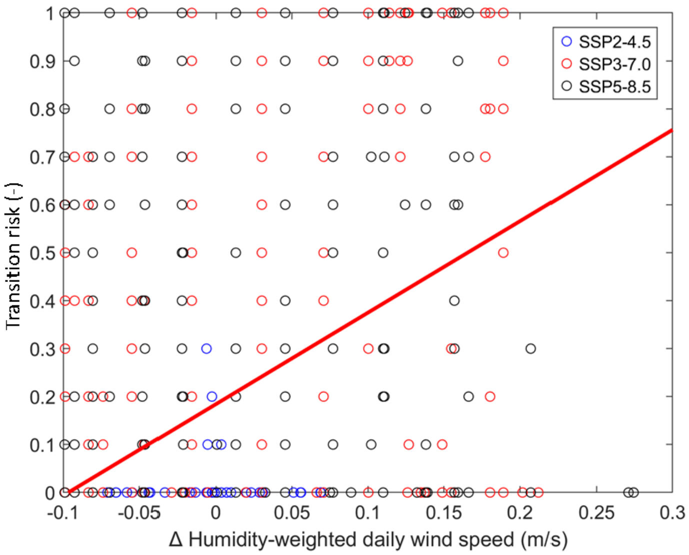

Figure S16 | Transition risks against humidity-weighted daily wind speeds. Transition risk in the 2090s across the three SSPs related to the change in humidity-weighted daily wind speed (m/s) in the respective SSP and decade compared to wind speed in SSP2-4.5 for 2021-2030. The data points represent the 416 cells on the NorESM2 grid, colored by SSP. The solid red line shows the linear regression, with R^2 = 0.33.

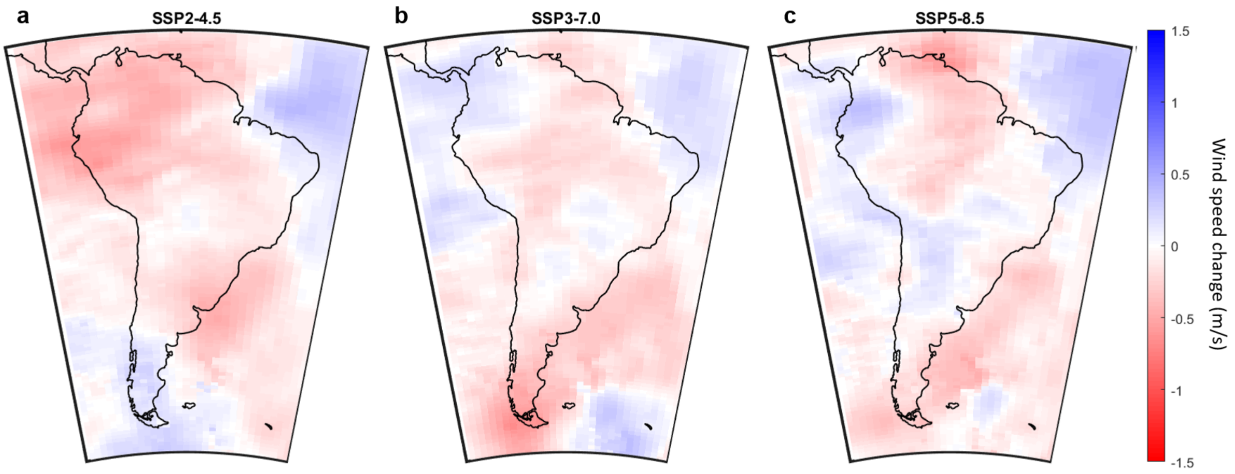

Figure S17| Spatial wind speed change for SSP scenarios. Average wind speed changes between the 2020s of SSP2-4.5 and the 2090s of each SSP for **a,** SSP2-4.5; **b,** SSP3-7.0; **c,** SSP585. Comparing this figure with Fig. 1 of the main manuscript indicates that wind speed changes are not the primary cause for tipping risks.

Optimal timestep for moisture tracking

The optimal length of timestep to use is the length at which most moisture parcels do not travel further than a single grid cell. To determine at which time step this is the case, the model was run for 2015 (SSP1-2.6) for the following time steps: 1h, 2h, 3h, 4h, 6h, 8h, 12h, and 24h, and the location of each parcel was saved at each timestep. The distances between these positions were then calculated, and the travel distance of a parcel was calculated (Fig. S18). In the Amazon, the spatial resolution of NorESM2 translates to 110 km by 111 km at the largest cells and 110 km by 104 km at the smallest cells. For a time step of 4 hours, the mean travel distance of a parcel in one timestep is 54.6 km with a standard deviation of 42.1 km. The mean plus one standard deviation is 7.3 km lower than the shortest possible grid cell dimension (104 km). Therefore, we have chosen 4 hours as the timestep for moisture tracking in this manuscript.

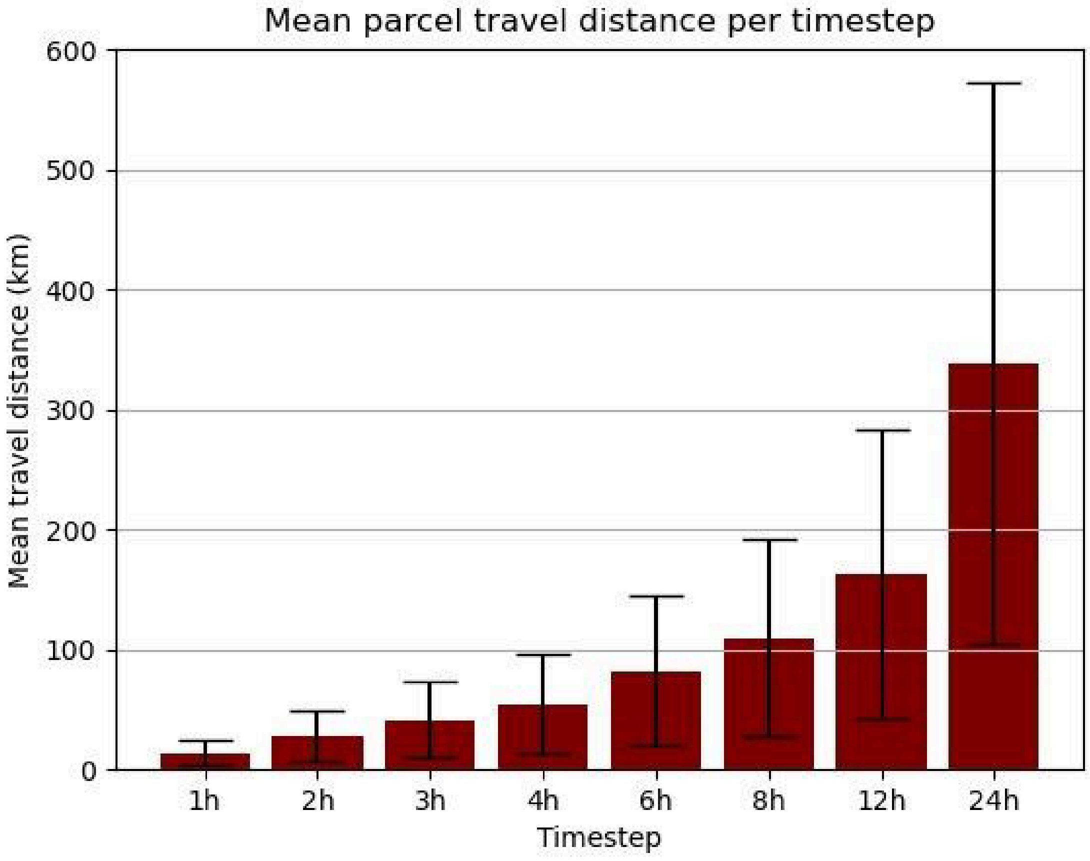

Figure S18 | Mean parcel travel distance per timestep. Mean $\pm$ one standard deviation of parcel travel distances in the Amazon per timestep of 1h, 2h, 4h, 6h, 8h, 12h and 24h in a run for 2015 (SSP1-2.6). Results are presented for 5 parcels which were tracked for 24 hours. The parcels were selected randomly across the Amazon basin.

Supplementary References

1. Soares-Filho, B. et al. LBA-ECO LC-14Modeled Deforestation Scenarios, Amazon Basin: 2002-2050. *ORNL DAAC, doi: 10.3334/ORNLDAAC/1153* (2013).
2. Flores, B. M. et al. Critical transitions in the amazon forest system. *Nature* **626**, 555–564 (2024).
3. Tuinenburg, O. A. & Staal, A. Tracking the global flows of atmospheric moisture and associated uncertainties. *Hydrol. Earth Syst. Sci.* **24**, 2419–2435 (2020).
4. Fricko, O. et al. The marker quantification of the Shared Socioeconomic Pathway 2: A middle-of-the-road scenario for the 21st century. *Global Environmental Change*, **42**, 251-267 (2017).
5. Staal, A., Koren, G., Tejada, G. & Gatti, L. V. Moisture origins of the Amazon carbon source region. *Environ. Res. Lett.* **18**, 044027 (2023).
6. Staal, A., Meijer, P., Nyasulu, M. K., Tuinenburg, O. A. & Dekker, S. C. Global terrestrial moisture recycling in Shared Socioeconomic Pathways. *Earth Syst. Dyn.* **16**, 215–238 (2025).
7. Jones, C., Mu, Y., Carvalho, L. M. & Ding, Q. The South America Low-Level Jet: form, variability and large-scale forcings. *npj Clim. Atmos. Sci.* **6**, 175 (2023).
