# Supplementary material for: Deforestation-induced drying lowers Amazon climate threshold
Source: Nature. 2026 May 6;654(8117):114–20. doi: 10.1038/s41586-026-10456-0 (PMC13233621; doi:10.1038/s41586-026-10456-0)
Supplement: Supplementary file 2 — Reporting Summary [file 41586_2026_10456_MOESM2_ESM.pdf]

nature portfolio

Corresponding author(s): *Double-anonymous peer review submissions: write DAPR and your manuscript number here instead of author names.*

Last updated by author(s): *YYYY-MM-DD*

Reporting Summary

Nature Portfolio wishes to improve the reproducibility of the work that we publish. This form provides structure for consistency and transparency in reporting. For further information on Nature Portfolio policies, see our Editorial Policies and the Editorial Policy Checklist.

Statistics

For all statistical analyses, confirm that the following items are present in the figure legend, table legend, main text, or Methods section.

n/a	Confirmed	
☐	☒	The exact sample size (*n*) for each experimental group/condition, given as a discrete number and unit of measurement
☐	☒	A statement on whether measurements were taken from distinct samples or whether the same sample was measured repeatedly
☒	☐	The statistical test(s) used AND whether they are one- or two-sided<br>*Only common tests should be described solely by name; describe more complex techniques in the Methods section.*
☒	☐	A description of all covariates tested
☒	☐	A description of any assumptions or corrections, such as tests of normality and adjustment for multiple comparisons
☐	☒	A full description of the statistical parameters including central tendency (e.g. means) or other basic estimates (e.g. regression coefficient) AND variation (e.g. standard deviation) or associated estimates of uncertainty (e.g. confidence intervals)
☒	☐	For null hypothesis testing, the test statistic (e.g. *F*, *t*, *r*) with confidence intervals, effect sizes, degrees of freedom and *P* value noted<br>*Give P values as exact values whenever suitable.*
☒	☐	For Bayesian analysis, information on the choice of priors and Markov chain Monte Carlo settings
☒	☐	For hierarchical and complex designs, identification of the appropriate level for tests and full reporting of outcomes
☒	☐	Estimates of effect sizes (e.g. Cohen's *d*, Pearson's *r*), indicating how they were calculated

Our web collection on statistics for biologists contains articles on many of the points above.

Software and code

Policy information about availability of computer code

Data collection	N/A
Data analysis	The code of this manuscript (also regarding data analysis) can be found at https://doi.org/10.6084/m9.775 figshare.28191128. The data analysis was further carried out based on the PyCascades software package version 1.0 (developed in Wunderling et al., 2021, European Physical Journal Sp. Topics, 1-14).

For manuscripts utilizing custom algorithms or software that are central to the research but not yet described in published literature, software must be made available to editors and reviewers. We strongly encourage code deposition in a community repository (e.g. GitHub). See the Nature Portfolio guidelines for submitting code & software for further information.

Data

Policy information about availability of data

All manuscripts must include a data availability statement. This statement should provide the following information, where applicable:

- Accession codes, unique identifiers, or web links for publicly available datasets
- A description of any restrictions on data availability
- For clinical datasets or third party data, please ensure that the statement adheres to our policy

The historical MAP and evaporation data as well as SSP-based land-use change scenarios are supplied under https://doi.org/10.6084/m9.775 figshare.28191128.

The moisture recycling data can be found in Staal et al. (2025, Earth System Dynamics, 16, 215-238) together with the respective SSP-based precipitation and evaporation data. The deforestation data can be accessed via the following doi: 10.3334/ORNLDAAC/1153.

Research involving human participants, their data, or biological material

Policy information about studies with human participants or human data. See also policy information about sex, gender (identity/presentation), and sexual orientation and race, ethnicity and racism.

Reporting on sex and gender	N/A
Reporting on race, ethnicity, or other socially relevant groupings	N/A
Population characteristics	N/A
Recruitment	N/A
Ethics oversight	N/A

Note that full information on the approval of the study protocol must also be provided in the manuscript.

Field-specific reporting

Please select the one below that is the best fit for your research. If you are not sure, read the appropriate sections before making your selection.

☐ Life sciences ☐ Behavioural & social sciences ☒ Ecological, evolutionary & environmental sciences

For a reference copy of the document with all sections, see nature.com/documents/nr-reporting-summary-flat.pdf

Ecological, evolutionary & environmental sciences study design

All studies must disclose on these points even when the disclosure is negative.

Study description	For the Amazon tipping experiments due to deforestation and global warming, 1.25 million ensemble members were generated (~3000 per grid cell in the study area of the Amazon basin) and run through the PyCascades:Amazon software framework leading to transition risk assessments.
Research sample	Precipitation, evaporation based on NorESM2, and moisture transport networks computed using the UT rack global hydrological model. Deforestation data publicly provided (see Data and Code availability statement).
Sampling strategy	Monte Carlo ensemble with 1.25 million ensemble members
Data collection	Earth System Model data that are publicly available
Timing and spatial scale	From 1950-2099 on a monthly timescale and a 1x1° grid basis for the study area (the Amazon basin).
Data exclusions	no exlusions
Reproducibility	For the five robustness checks (see methods section "Robustness checks"), the data needed to be prepared individually, and also the code was prepared and checked separately. Through these separate test, high confidence in reproducibility is given. Further, regarding data and code availability, see the respective statement above.
Randomization	Random sampling (see methods section "Ensemble construction")
Blinding	N/ A because random ensemble was generated

Did the study involve field work? ☐ Yes ☒ No

Reporting for specific materials, systems and methods

We require information from authors about some types of materials, experimental systems and methods used in many studies. Here, indicate whether each material, system or method listed is relevant to your study. If you are not sure if a list item applies to your research, read the appropriate section before selecting a response.

Materials & experimental systems

n/a	Involved in the study
☒	☐ Antibodies
☒	☐ Eukaryotic cell lines
☒	☐ Palaeontology and archaeology
☒	☐ Animals and other organisms
☒	☐ Clinical data
☒	☐ Dual use research of concern
☒	☐ Plants

Methods

n/a	Involved in the study
☒	☐ ChIP-seq
☒	☐ Flow cytometry
☒	☐ MRI-based neuroimaging

Plants

Seed stocks	N/A
Novel plant genotypes	N/A
Authentication	N/A
